# Hydrogels Associated with Photodynamic Therapy Have Antimicrobial Effect against *Staphylococcus aureus*: A Systematic Review

**DOI:** 10.3390/gels10100635

**Published:** 2024-09-30

**Authors:** Ricardo S. Moura, João Pedro R. Afonso, Diego A. C. P. G. Mello, Renata Kelly Palma, Iransé Oliveira-Silva, Rodrigo F. Oliveira, Deise A. A. P. Oliveira, Dante B. Santos, Carlos Hassel M. Silva, Orlando A. Guedes, Giuseppe Insalaco, Luís V. F. Oliveira

**Affiliations:** 1 Human Movement and Rehabilitation, Graduate Program, Evangelical University of Goiás—UniEVANGÉLICA, Anápolis 75083-450, GO, Brazil; ricardos_moura@hotmail.com (R.S.M.); joaopedro180599@gmail.com (J.P.R.A.); diego0611escs@hotmail.com (D.A.C.P.G.M.); rekellyp@hotmail.com (R.K.P.); iranseoliveira@hotmail.com (I.O.-S.); rodrigofranco65@gmail.com (R.F.O.); deisepyres@gmail.com (D.A.A.P.O.); dantebsantos@gmail.com (D.B.S.); carloshmendes@unievangelica.edu.br (C.H.M.S.); 2 Facultad de Ciencias de la Salud de Manresa, Universitat de Vic-Universitat Central de Catalunya (UVic-UCC), 08243 Manresa, Spain; 3 Graduate Master’s Degree Program in Dentistry, Evangelical University of Goiás—UniEVANGÉLICA, Anápolis 75083-450, GO, Brazil; orlandoaguedes@gmail.com; 4 Institute of Translational Pharmacology, National Research Council of Italy (CNR), 90146 Palermo, Italy; giuseppe.insalaco@ift.cnr.it

**Keywords:** hydrogels, photodynamic therapy, antimicrobial effects, *Staphylococcus aureus*

## Abstract

*Staphylococcus aureus* (*S. aureus*) is a Gram-positive bacterium that causes infections ranging from mild superficial cases to more severe, potentially fatal conditions. Many photosensitisers used in photodynamic therapy are more effective against superficial infections due to limitations in treating deeper tissue infections. Recently, attention to this bacterium has increased due to the emergence of multidrug-resistant strains, which complicate antibiotic treatment. As a result, alternative therapies, such as antimicrobial photodynamic therapy (PDT), have emerged as promising options for treating non-systemic infections. PDT combines a photosensitiser (PS) with light and oxygen to generate free radicals that destroy bacterial structures. This systematic review evaluates the effectiveness of PDT delivered via different types of hydrogels in treating wounds, burns, and contamination by *S. aureus*. Following PRISMA 2020 guidelines, a bibliographic search was conducted in PubMed, Web of Science, and Scopus databases, including articles published in English between 2013 and 2024. Seven relevant studies were included, demonstrating evidence of PDT use against *S. aureus* in in vitro and in vivo studies. We concluded that PDT can effectively complement antimicrobial therapy in the healing of wounds and burns. The effectiveness of this technique depends on the PS used, the type of hydrogel, and the lesion location. However, further in vivo studies are needed to confirm the safety and efficacy of PDT delivered via hydrogels.

## 1. Introduction

The rise of antimicrobial resistance in bacteria, viruses, fungi, and parasites presents a significant global challenge to human health and development [1]. The World Health Organisation recognises it as one of the main concerns for global public health, ranking among the top ten threats to humanity [1]. Inappropriate and excessive use of antimicrobial agents contributes to resistance, making practically all pathogenic microorganisms insensitive to the medications commonly used to control them. The rise in multidrug resistance to key antibiotic classes has led to a surge in hospital-acquired pathogens. These include *Enterococcus faecium*, *Staphylococcus aureus*, *Klebsiella pneumoniae*, and Enterobacter species, collectively referred to as the ESKAPE group [2]. Despite the complexity of developing new drugs with antibiotic properties [1], alternative approaches have been developed recently to control the spread of pathogenic microorganisms. One such approach is antimicrobial photodynamic therapy (PDT), which uses photosensitising agents (dyes) and hydrogels capable of absorbing light photons and elevating them to an excited state [3].

In recent years, the rise of drug-resistant bacteria has made existing antibiotics less effective, while the development of biofilms has further diminished their therapeutic impact [4]. The massive and abusive use of antibiotics has caused severe side effects, making it imperative to develop alternative, ultra-efficient, and safe antibacterial systems [4]. The most recent technique used to control microbial infections is the combination of PDT with a hydrogel, which appears innovative and operates non-specifically in microbial cells, thus preventing the development of resistance [5].

Photodynamic therapy (PDT) involves administering a non-toxic, light-sensitive photosensitiser (PS), which is then activated by exposure to light, typically using doses that are carefully adjusted to the clinical application and type of PS used. Upon activation by light, and in the presence of oxygen, the PS can undergo a series of reactions leading to the production of reactive oxygen species (ROS). These reactions include the transfer of electrons or hydrogen to nearby molecules, resulting in the formation of free radicals, such as superoxide radicals and hydroxyl radicals, or energy transfer to oxygen, producing singlet oxygen (^1^O_2_). Both pathways result in the generation of ROS that can induce cell death and destruction of diseased tissues [5].

Faced with the growing problem of resistance to microbial antibiotics, PDT delivered using hydrogels has attracted interest as an alternative antimicrobial treatment. Several in vitro and in vivo studies involving microbial inactivation with successful results for bacteria, fungi, yeasts, viruses, and parasites, and in the healing of wounds and burns have been conducted [6]. Furthermore, a recent study has demonstrated that photosensitisation of bacterial cells is independent of the spectrum of antibiotic resistance [5].

The photosensitiser (PS) must be selected so that the available light source provides photons at the appropriate absorption wavelength for effective activation [7]. The penetration of light through tissue can be hindered by dispersion and absorption, which depend on the wavelength of the incident light, the type of tissue, and how the PS is delivered to the area to be decontaminated. Hydrogels are excellent carriers of PSs for photodynamic therapy (PDT) [5]. Hydrogels have demonstrated good performances as cell carriers with different functionalities [8].

Hydrogels are three-dimensional polymer networks expanded with water, offering adjustable physicochemical properties that meet various needs across different conditions. These versatile materials have found extensive use in biomedicine, ranging from investigating physiological and pathological mechanisms to applications in tissue regeneration and disease therapy [9].

Hydrogels have been widely investigated as matrices for biomedical applications because of their crosslinking ability under mild conditions, excellent biocompatibility, and tunable biochemical and biophysical properties. As their structure and properties resemble the microenvironment of many tissues in the human body, they are widely used in various biomedical applications [10].

In summary, multifunctional and intelligent antibacterial hydrogels designed according to the actual needs can simultaneously provide broad prospects for antibacterial infection therapy and tissue reconstruction. This study aimed to conduct a systematic review of studies analysing the effectiveness of PDT delivered via hydrogels for treating infections caused by *S. aureus*.

## 2. Materials and Methods

### 2.1. Development

This systematic review followed the Preferred Reporting Items for Systematic Reviews and Meta-Analyses (PRISMA) 2020 guidelines [11] (Registration ID 586572). Bibliographic research was conducted using the PubMed, Web of Science, and Scopus databases. Only full articles published in English between 2013 and 2024 from any country of origin (without restrictions) were included. The research was conducted from 15 January 2024 to 15 June 2024 and did not use any automatic bibliographic search tool.

### 2.2. Data Extraction Process

A two-step process was used to select studies. In the first phase, two reviewers (RSM and JPRA) independently screened the titles and abstracts to identify studies that met the eligibility criteria. Studies that satisfied these criteria were chosen for full-text review. In the second phase, the same reviewers independently assessed the full texts to confirm their inclusion. Any disagreements between the two reviewers were resolved through discussion with a third reviewer (LVFO), if needed.

For each selected database, a bibliographic search was performed using titles and abstracts, with keywords aligned with MeSH terms. The search strategy involved a predefined combination of keywords as follows (Table 1):

“Hydrogels” [Mesh] OR Hydrogel OR In Situ Hydrogels OR In Situ Hydrogel OR Patterned Hydrogels OR Patterned Hydrogel “Photochemotherapy” [Mesh] OR Photochemotherapies OR Photodynamic Therapy OR Photodynamic Therapies “Gram-Positive Bacterial Infections” [Mesh] OR Gram Positive Bacterial Infections OR Gram-Positive Bacterial Infection “*Staphylococcus aureus*” [Mesh]”Anti-Infective Agents” [Mesh] OR Anti Infective Agents OR Antiinfective Agents OR Anti-Infective Agent OR Anti Infective Agent OR Microbicides OR Anti-Microbial Agent OR Anti Microbial Agent OR Antimicrobial Agents OR Anti-Microbial Agents OR Anti Microbial Agents OR Microbicide OR Antimicrobial Agent.

All the titles were manually searched and reviewed for inclusion. Reference lists of the articles containing titles, author names, languages, and publication dates were generated. This systematic review included only scientific articles reporting experimental studies.

### 2.3. Election Criteria

#### 2.3.1. Design and Interventions

This review examined controlled experimental laboratory studies that employed PDT in conjunction with different hydrogels to combat antibiotic-resistant microorganisms, particularly *S. aureus*. Both in vitro and in vivo experimental models were considered, including research conducted on animals and studies that investigated the effects of PDT on cells without the use of animals.

#### 2.3.2. Methodological Design

For this scoping review, we queried PubMed (using MeSH), Web of Science, and Scopus databases to identify relevant articles. We used specific search criteria, including keywords such as *Staphylococcus aureus*, photodynamic therapy, photosensitiser, Gram-positive, hydrogel, and antibiotic resistance. The inclusion and exclusion criteria were applied to ensure the relevance of the articles. We included studies that explored the photodynamic activity associated with antimicrobial effects against *S. aureus*, both in vitro and in vivo, considering its clinical applications and synergy with other antimicrobial agents. We excluded unpublished research, studies published before 2013, studies that did not mention the PS used, those that did not involve *S. aureus*, those that did not use hydrogels in PDT, and those that were not clinically relevant to the research. We followed the PRISMA guidelines, which allowed for a systematic approach to article selection. After selecting the studies, data were extracted to ensure consistency in the inclusion and exclusion criteria.

## 3. Results

### 3.1. Study Description

**Figure 1:**
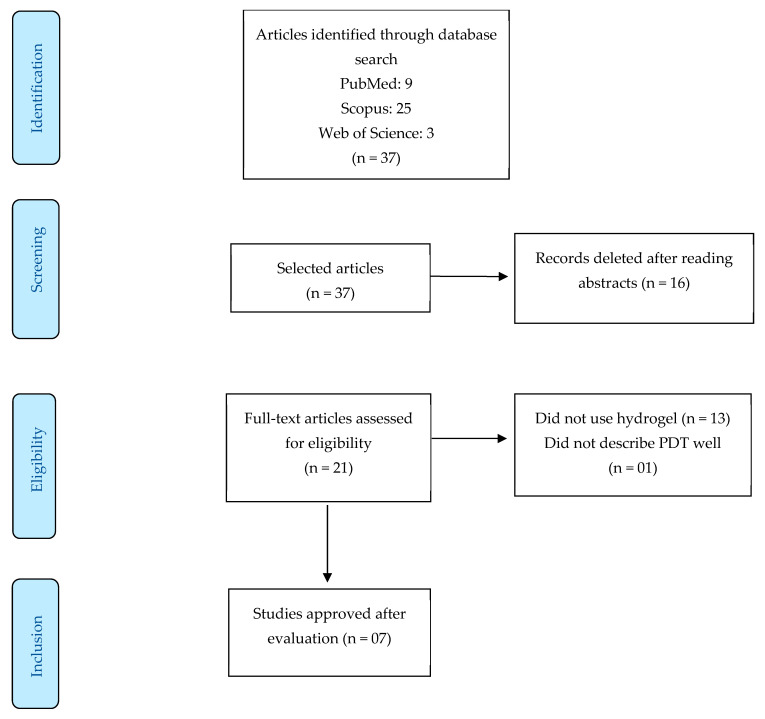
Flow diagram of the current systematic review conducted according to the Preferred Reporting Items for Systematic Reviews and Meta-analysis (PRISMA) guidelines.

The initial search found 37 articles, which, after being subjected to the evaluation criteria, left only seven articles that addressed the topic of this study (Figure 1). Table 2 summarises each article and its particularities.

### 3.2. Characteristics and Results of Individual Studies

#### 3.2.1. Xylan–Porphyrin Hydrogels as Light-Triggered Gram-Positive Antibacterial Agents

This study describes the synthesis and characterisation of Xylan-based hydrogels containing a PS (tetra(4-carboxyphenyl) porphyrin; TCPP) for in vitro bacterial photoinactivation. The main results and conclusions are as follows. Hydrogels were prepared by crosslinking Xylan with TCPP using N, N′-carbonyldiimidazole as a coupling agent. Different amounts of TCPP were used to synthesise the hydrogels [15].

Freeze-drying of the hydrogels affected their ability to swell in water, and shrinkage promoted mutual hydrophobic interactions, making it difficult for water to re-enter the hydrogel structure. Hydrogels containing lower amounts of porphyrins (TCPP) exhibited better swelling properties than those containing higher amounts. Covalent bonding between TCPP and Xylan was confirmed through Fourier transform infrared (FTIR) spectroscopy [17]. In vitro bacterial photoinactivation tests showed that the hydrogel functionalised with TCPP exhibited antibacterial activity only under light irradiation and was more effective against Gram-positive bacteria. The concentration of TCPP in the hydrogel affects the antibacterial activity, and further research is needed to optimise the concentration of the PS [15].

This research demonstrates the successful synthesis of TCPP-containing Xylan-based hydrogels that show potential for bacterial photoinactivation applications, particularly against Gram-positive bacteria. However, the optimal concentrations of TCPP and other parameters need to be determined.

#### 3.2.2. Optimisation and Evaluation of a Chitosan/Hydroxypropylmethylcellulose Hydrogel Containing Toluidine Blue for Antimicrobial Photodynamic Inactivation

This study by Brown et al. (1993) describes the formulation and characterisation of hydrogels containing chitosan (HCT) as potential agents for PDI against *S. aureus* and *P. aeruginosa* biofilms. Hydroxypropylmethylcellulose (HPMC) was used as a gelling agent, and the effects of different concentrations of HPMC on the physical and textural properties of hydrogels, including viscosity, hardness, adhesiveness, and compressibility, were evaluated [18]. Chen et al. (2015) showed that increasing the concentration of HPMC in the HCT resulted in greater viscosity, hardness, adhesiveness, and compressibility. The hydrogel with 1% HPMC exhibited textural properties similar to those of a commercial gel. However, the injectability of the hydrogels decreased as the concentration increased [12]. The efficacy of PDI against *S. aureus* and *P. aeruginosa* biofilms was evaluated, and the results show that HCT with low concentrations of HPMC (F-1 and F-2) were effective, similar to those of the mixture of toluidine blue O (TBO) and chitosan. However, increasing the HPMC concentration reduced the effectiveness of PDI, probably due to the restriction of TBO release in the hydrogels with high viscosity. Furthermore, another study investigated the penetration of TBO into biofilms and observed that increasing the concentration of HPMC restricted the penetration of TBO into the deeper layers of the biofilms [19]. In an in vivo study, HCT were tested in a burn model of rat skin infected with *S. aureus*, and the results show a significant reduction in the survival of bacterial cells. However, the effectiveness of this treatment decreased as the HPMC concentration in the hydrogel increased. Overall, this study highlights the importance of HPMC concentration in the formulation of hydrogels for PDI and the need to optimise textural properties and treatment efficacy [12].

#### 3.2.3. Hydrogen Peroxide (H_2_O_2_)-Supramolecular Material for the Treatment of Post-Irradiation Infected Wounds

This article presents a study on the production of H_2_O_2_ through a photocatalytic process mediated by riboflavin and its use for antibacterial purposes. The research is based on several steps and discoveries, as described below:

1. Photocatalytic Process and H_2_O_2_ Production: Riboflavin was used as a photocatalyst and had a strong absorption peak around 460 nm. After irradiation with blue light, riboflavin was excited and rapidly converted into a triple state with a high oxidation potential, generating H_2_O_2_. The amount of H_2_O_2_ was quantified by monitoring the changes in the absorbance at 652 nm [20].
2. Choice of Guanosine: Among the nucleotides derived from guanine, guanosine generates the most substantial amount of H_2_O_2_ owing to hydrogen bonds and stacking interactions with riboflavin. Because of the differences in their oxidation potentials, guanosine produces more H_2_O_2_ than adenosine, uridine, or cytidine [21].
3. G4 Supramolecular Materials: Guanosine was used to develop G4 supramolecular materials, which were formed into nanofibres and crosslinked using 4-formylphenylboronic acid and 1,8-diaminooctane. The properties of these materials were characterised using techniques such as electrospray mass spectrometry and FTIR spectroscopy [8]. It is very interesting to highlight the role of guanosine in the formation of G-quartets, which are essential for the structural integrity and function of certain photoactive materials. These G-quartets, in combination with riboflavin, facilitate the production of ROS, including H_2_O_2_, which increases the antibacterial efficacy of the treatment.
4. Controlled H_2_O_2_ Production: The amount of H_2_O_2_ generated can be controlled by varying the riboflavin concentration and irradiation time. This system maintains its robustness even after irradiation.
5. Antibacterial Activity: The H_2_O_2_ generated was used to test the antibacterial activity. The post-irradiation riboflavin-loaded hydrogel effectively killed Gram-positive, Gram-negative, and multidrug-resistant bacteria with a sterilisation rate of over 99.999%. Incubation with the catalase inhibited the antibacterial activity [22].
6. In Vivo Assays: The study included in vivo assays using an MRSA-infected rat wound model. The post-irradiation hydrogel exhibited a significant therapeutic effect by eliminating wound infections and reducing the levels of typical inflammatory factors. This study demonstrates the effectiveness of controlled riboflavin-mediated H_2_O_2_ production for antibacterial purposes with promising results both in vitro and in vivo [23].

#### 3.2.4. Photo-Inspired Antibacterial Activity and Acceleration of Wound Healing by Hydrogel Incorporated with Ag/Ag@Siver Chloride (AgCl)/Zinc Oxide (ZnO) Nanostructures

This study presents the synthesis and characterisation of a nanocomposite hydrogel containing Ag/Ag@AgCl/ZnO. The hydrogel was obtained by absorbing water and opening pores in the gel structure. X-ray diffraction (XRD) results revealed changes in the hydrogel structure after doping with Ag/Ag@AgCl/ZnO, indicating the incorporation of these nanomaterials. The diffraction patterns showed peaks corresponding to the crystal planes of metallic Ag, AgCl, and ZnO [24]. The morphology of the pure hydrogel was similar to that of a sponge, with pores of approximately 10 μm in diameter. After doping with the Ag NPs and Ag@AgCl particles, the Ag NPs were uniformly distributed in the samples. The ZnO hydrogel incorporated one-dimensional ZnO nanostructures, such as nanorods and aggregates. The presence of Ag NPs in the ZnO nanostructures was confirmed using microscopy [25]. An analysis of the release of Ag and Zn ions from the hydrogels revealed different release profiles. The antibacterial activities of the hydrogels were evaluated in vitro, and the results showed significant effects against bacteria. Reactive oxygen species (ROS) formation was detected and was associated with enhanced antibacterial activity. In vivo studies demonstrated the therapeutic efficacy of hydrogels for wound healing, particularly in wounds infected with *S. aureus*. The Ag/Ag@AgCl/ZnO and pure ZnO hydrogels reduced bacterial infection and promoted wound healing [14].

This article introduces a novel approach for the synthesis of nanocomposite hydrogels that exhibit promising antibacterial properties. This study also highlights the potential of these hydrogels for wound healing, particularly in the context of infected wounds. The unique properties of these hydrogels, such as the controlled release of metal ions and generation of ROS, contribute significantly to their antibacterial and therapeutic efficacy.

#### 3.2.5. Carrageenan Embedded in Atomically Precise Au Nanocluster for Single Infrared Light-Driven Photothermal and Photodynamic Antibacterial Therapy

The study addresses the synthesis and characterisation of a nanocomposite hydrogel composed of Ag, Ag@AgCl, and ZnO [26]. The main points of the study are as follows. The synthesis of the nanocomposite hydrogel involves the absorption of water by the carboxymethyl cellulose hydrogel, followed by doping with Ag/Ag@AgCl/ZnO. The materials were characterised via XRD to analyse their crystalline structures [27]. The results indicate the presence of metallic Ag, AgCl, and ZnO in the hydrogel structure. Transmission electron microscopy images showed the presence of cubic metallic Ag and Ag@AgCl nanostructures along with one-dimensional ZnO nanostructures [5]. The swelling behaviour of the hydrogel was investigated at different pH values, and it was found to be pH-sensitive [28]. The study also evaluated the release of Ag^+^ and Zn^2+^ ions from the nanocomposite hydrogel and its antibacterial activity in vitro. These results indicate that the nanocomposite hydrogel had antibacterial properties with different effects against *Escherichia coli* and *S. aureus* [29].

This study also evaluated the cytotoxicity of these materials in cell culture and their effectiveness in wound healing in an animal model. The results show that the nanocomposite hydrogel could potentially be used for the treatment of infections and wound healing. Overall, this study focused on synthesising and characterising a nanocomposite hydrogel, highlighting its antibacterial properties and potential for wound healing [5].

#### 3.2.6. Optimisation of Hydrogel Containing Toluidine Blue for PDT in the Treatment of Acne

This study focused on optimising the formulation of a hydrogel containing the PS, TBO, for PDT to treat bacterial infections, particularly acne vulgaris. Four types of carbomers were used, namely, TBO, Tween 80, and other chemicals. PDT was performed on different types of bacteria, including *S. aureus*, *E. coli*, and *Propionibacterium acnes*, using the TBO hydrogel as a PS [26].

The results demonstrate that various factors, including the type of carbomer, carbomer concentration, TBO concentration, ethanol concentration, Tween 80 ratio, and mass ratio of NaOH to carbomer, significantly influenced the effectiveness of the TBO hydrogel in photodynamic therapy (PDT). The optimal hydrogel formulation was determined through optimisation experiments using response surface methodology (RSM) [30]. This formulation comprised 0.5% (*w*/*v*) carbomer 934, 0.01 mg/mL TBO, 0.5% (*v*/*v*) ethanol, 0.5% (*v*/*v*) Tween 80, and a 0.4 (*c*/*c*) mass ratio of NaOH to carbomer. This hydrogel exhibited excellent rheological properties, stability during storage, and antibacterial activity against the tested bacteria [5]. When compared with the optimal TBO hydrogel PDT and antibiotic therapy, the PDT showed promising results in reducing colony-forming units/mL. The release of TBO from the hydrogel was also evaluated, showing that the optimised formulation maintained its properties over time, in terms of pH and viscosity. This study shows that PDT with the optimised TBO hydrogel is a promising approach for treating bacterial infections, such as acne vulgaris, and may represent an effective alternative to traditional antibiotics [28]. The optimisation process involves adjusting parameters, such as light dose, photosensitiser concentration, and irradiation time, to maximise the loss of viable microorganisms while ensuring safety and minimal side effects.

#### 3.2.7. Optimisation of Hydrogel Containing Toluidine Blue for PDT Using RSM

This study involved the preparation of hydrogels containing TBO and their application in the inactivation of *S. aureus* and *E. coli* through antimicrobial photodynamics (PDT) [31]. The materials used included carbomers, TBO, NaOH, and other reagents. The hydrogels were prepared by mixing the components and optimising the concentrations of carbomer, TBO, and the quality ratio between NaOH and carbomer using RSM [13].

The results show that the choice of carbomer type did not significantly affect the antimicrobial activity. However, the carbomer concentration influenced the activity, with very high concentrations impairing the diffusion of TBO in bacterial cells. The TBO concentration also affected the activity, with a 0.1 mg/mL concentration chosen for further experiments. The ratio of NaOH to carbomer also influenced the antimicrobial activity, with a ratio of 0.4 found to be the most effective [13].

A single-factor and RSM experiment identified an ideal hydrogel formulation comprising 3% carbomer, 0.1 mg/mL TBO, and a quality ratio of NaOH and carbomer of 0.4. This formulation exhibited potent antibacterial activity against *S. aureus* and *E. coli*. This study also evaluated the stability of the hydrogel, which remained stable during 6 weeks of storage at different temperatures. Furthermore, the release of TBO from the hydrogel was monitored, and it showed gradual release over 6 h [32].

It is essential to highlight that the application of the TBO hydrogel or light alone did not result in significant antibacterial activity, highlighting the importance of combining both for PDT. In summary, this study demonstrates an ideal formulation of a hydrogel containing TBO with high antibacterial activity and good stability. Thus, PDT is a promising candidate for clinical treatments involving the inactivation of bacteria such as *S. aureus* and *E. coli* [31].

## 4. Discussion

### 4.1. Main PSs

#### 4.1.1. Methylene Blue (MB)

MB, a phenothiazine, is commonly utilised as a photosensitiser (PS) for photodynamic therapy (PDT) due to its high efficiency in producing singlet oxygen (^1^O_2_). In addition to its photodynamic properties, MB possesses intrinsic antimicrobial activity that enhances its light absorption capabilities [33]. Effective across a wavelength range of 625–635 nm, MB targets both Gram-positive and Gram-negative bacteria. Its effectiveness as a PS is partly due to its hydrophilic/lipophilic balance and its strong affinity for cellular membranes, which facilitates its penetration through plasma membranes and the generation of intracellular reactive oxygen species (ROS) upon light activation [34]. MB has long been recognised for its use as a histological stain and antiseptic dye, and it has been employed as a topical disinfectant for wounds and infections for many years. Furthermore, it is one of the most commonly used PSs in photodynamics, alone or in combination with other compounds, such as nanoparticles (NPs) or antibiotics [35].

Previous studies have demonstrated the synergistic effects of MB in combination with ethanol and ethylenediaminetetraacetic acid (EDTA). The addition of these components significantly inhibits bacterial growth, suggesting a synergistic action. While ethanol can prolong singlet oxygen production during PDT, potentially increasing its effectiveness, its use may also denature proteins, affecting both light and photosensitiser penetration. EDTA disrupts biofilms and damages bacterial outer membranes, facilitating the transport and absorption of ethanol and MB molecules within the bacteria, which leads to increased singlet oxygen production and enhanced PDT efficacy [7]. Although optimising PDT with ethanol shows promise, its clinical application in treating superficial wounds remains debated. Therefore, it is crucial to ensure that optimising PDT with ethanol is clinically safe, particularly in wound care settings [36].

#### 4.1.2. Rose Bengal (RB)

RB, first identified by Gnehm, is a water-soluble anionic xanthene dye and a halogenated derivative of fluorescein [37]. It consists of three aromatic rings arranged linearly with an oxygen atom in the centre and functions as a type II photosensitiser [37]. When activated by visible light, RB exhibits maximum absorption at 546 nm in water [38]. Due to its anionic nature, RB may be less effective against Gram-negative bacteria, such as *Pseudomonas aeruginosa*, because of the negative charges on the bacterial membrane. Strategies to improve RB’s effectiveness include its incorporation into cationic polymers, both of natural and synthetic origin, which act as carriers to enhance its antimicrobial efficacy [39].

A study by Fernandez et al. (2021) demonstrated that the interaction of RB with potassium iodide in a Poly 2-hydroxyethyl methacrylate (PHEMA) matrix creates a protective environment for the photosensitiser (PS), preventing photodegradation and prolonging its half-life. This strategy reduced the light dose required for PDT while effectively eradicating planktonic cells. PHEMA, known for its transparency, is widely used in medicine for manufacturing contact lenses and urethral stents [40].

Another approach involves combining RB with polycationic chitosan, a natural biopolymer with high antimicrobial activity, biocompatibility, and biodegradability. This combination showed promising bacterial eradication when activated by light, suggesting its potential for clinical applications. Additionally, combining RB with cation exchange resins, like macroporous polystyrene and Amberlite® IRA-900 (Wilmington, DE, USA), resulted in significant inhibition of bacterial viability. This interaction increased efficacy against Gram-negative bacteria, such as *P. aeruginosa*, with cationic transporters overcoming RB’s initial ineffectiveness against these species [41]. These strategies highlight RB’s versatility and potential for optimising PDT to treat resistant infections.

#### 4.1.3. Porphyrins

Porphyrins are a group of fluorescent crystalline pigments, either naturally occurring or synthetically derived, that are widely used in PDT. They have notable absorption bands around 392 nm (Soret band) and weaker satellite absorptions between 495 and 616 nm (Q bands) [42]. In living tissues, porphyrins play vital roles in processes such as oxygen transport and photosynthesis [43].

The efficacy of porphyrins in PDT can be compromised by their difficulty in penetrating Gram-negative bacterial cell membranes due to the negatively charged cell wall. To overcome this, cationic porphyrins that interact effectively with negatively charged bacterial structures have been developed. For neutral or anionic porphyrins, this barrier can be circumvented with membrane-disrupting agents or by attaching cationic polypeptides to the PS molecules [44].

A prominent cationic porphyrin, 5,10,15,20-tetrakis(1-methylpyridinium-4-yl) (TMPyP), has shown significant antimicrobial activity against *P. aeruginosa*. TMPyP’s photocationic properties enable strong electrostatic interactions with negatively charged bacterial surfaces. When nanoassembled with cyclodextrin (CAPTISOL) as a carrier, TMPyP demonstrates enhanced penetration of the bacterial plasma membrane. The TMPyP/CAPTISOL combination exhibits excellent stability and photostability in biologically relevant environments and as freeze-dried solids. Furthermore, this combination is effective at eliminating 99% of bacteria, functioning as a sustained-release photosensitiser with enhanced stability compared to the free compound, making it a promising candidate for intravenous administration and pre-surgical applications [45].

However, despite TMPyP’s effectiveness, its absorbance spectrum at 630 nm and nearby wavelengths may limit its tissue penetration depth. For deeper tissue applications, photosensitisers with longer wavelength absorption, such as chlorins and bacteriochlorins, may offer superior performance by penetrating deeper and reducing scattering at shorter wavelengths. These alternatives could potentially address the limitations of TMPyP’s absorption properties.

#### 4.1.4. Riboflavin (RF)

Riboflavin, also known as vitamin B2, is a natural, non-toxic photosensitiser (PS) with various applications, including the decontamination of blood, plasma, or cell extracts and the elimination of microorganisms when combined with UV irradiation [46]. Additionally, riboflavin is inexpensive, highly biocompatible, and can be activated by light-emitting diode (LED) lamps in the ultraviolet A (360 nm) and blue (440 nm) regions [47]. This ability to be activated by visible light expands its applications and makes it a non-toxic photoinitiator.

Recently, riboflavin has been used as a photoinitiator in the preparation of hydrogels exposed to visible light and is recognised as a biocompatible photocrosslinking agent. Furthermore, when associated with a light-emitting source, such as an LED, it can act as a sterilising agent [48]. Its water solubility and biocompatibility make riboflavin widely used in the biomedical field [46].

It is important to note that riboflavin has effective absorption in the blue (440 nm) and UV-A (360 nm) regions, which is suitable for many PDT applications despite the limited light penetration at shorter wavelengths. Although riboflavin may not be the most effective for deeper tissue penetration, its ability to generate reactive oxygen species (ROS), such as H_2_O_2_, makes it useful for superficial applications. Riboflavin indeed absorbs most effectively in the UV-A and blue regions (360 nm and 440 nm). However, its utility in photodynamic therapy (PDT) primarily leverages its ability to generate reactive oxygen species (ROS) under visible light irradiation, even if the penetration depth is limited. While riboflavin may not have the ideal absorption spectrum for deep tissue penetration, its high biocompatibility and ability to produce H_2_O_2_ and other ROS make it effective for surface-level or superficial applications. Upon irradiation, riboflavin can indeed generate H_2_O_2_ as a major reactive oxygen species. The production of H_2_O_2_ plays a significant role in the antibacterial activity of riboflavin-mediated PDT, especially in applications where deeper penetration is not necessary. Notably, the amount of riboflavin required for hydrogel formation is small, generally less than 5 mg per 1 g of hydrogel precursor. Thus, riboflavin as a photoinitiator for hydrogel manufacture is considered safe and even beneficial. Moreover, an overdose of riboflavin does not cause significant side effects, as excess riboflavin is excreted in the urine a few hours after ingestion [8]. The visible light absorption spectrum of riboflavin allows for the initiation of photopolymerisation through visible light irradiation [49].

### 4.2. PDT in Clinical Isolates

PDT studies involving clinical isolates of *P. aeruginosa* are relatively recent, dating back to 2019. A previous study [17] used clinical isolates from wounds infected with *P. aeruginosa* and other ESKAPE strains from Leipzig Hospital. The researchers demonstrated the complete eradication of *P. aeruginosa* clinical isolates through the application of two porphyrin-based PSs, TMPyP and THPTS, embedded in a hydrogel matrix. TMPyP was activated at a wavelength of 420 nm and a light dose of 13 mW/cm^2^, while THPTS was activated at a wavelength of 420 nm and a light dose of 18 mW/cm^2^ [50].

One of the fundamental objectives of this study was to explore the use of these hydrogels as adhesives or wound dressings to relieve pain, promote adequate wound healing, and absorb exudates. Translucent hydrogels have been highlighted as an excellent choice, as they allow the effective application of doses and wavelengths of light without the need to remove the substrate. This characteristic facilitates the practical and effective application of PDT, similar to other studies on chitosan hydrogels [17].

Additionally, RM24, one of the photosensitisers used, showed significantly greater bactericidal activity against strains in the exponential growth phase compared to those in the steady state, even at concentrations as low as 1 µM. The researchers also noted that the presence of organic compounds can impact the effectiveness of RM24, suggesting that the medium influences the activity of photosensitisers [51].

Sodium azide was used as the antioxidant to characterise the ROS produced by the activation of RM24. However, notably, the use of this highly toxic compound in photodynamics is currently not recommended, highlighting the importance of safety considerations in this type of research [52].

### 4.3. Synergism with Antibiotics and Other Drugs

Many studies have explored the combination of PSs and antibiotics to optimise the efficacy of PDT against *P. aeruginosa*. Some notable examples are as follows:

1. Amoxicillin with Gold NPs (amoxi@AuNPs): The combination of gold nanoparticles (AuNPs) as a photosensitiser (PS) and amoxicillin has demonstrated significant potential in inhibiting the growth of *Pseudomonas aeruginosa*. When activated with white LED light at 490 nm for 3 h, this combination achieved a bacterial load reduction of over 70%, equivalent to approximately 1.5 cell divisions. This synergistic approach not only enhances the antibacterial efficacy but also suggests a strategy to reduce the reliance on high doses of antibiotics, thereby minimising their adverse effects. Notably, the use of amoxicillin with AuNPs reduced the required light activation time, making photodynamic therapy (PDT) more practical and efficient. The light dose used in these experiments was precisely calculated to ensure optimal activation of the PS, and the effectiveness was assessed in terms of the reduction in colony-forming units (CFU/mL). This combined approach leverages the photodynamic properties of gold nanoparticles while enhancing the antibacterial action of amoxicillin, offering a promising alternative to traditional antibiotic therapies [34].
2. MB with Gentamicin (Gen + MB): One study used a combination of MB and gentamicin for PDT against *P. aeruginosa*. Red LED light resulted in a notable inhibition of 6 log cm^2^ in planktonic cultures and 3 log cm^2^ in biofilms. The addition of gentamicin reduced the amount of methylene blue required for photoactivation, indicating potential advantages for the treatment of skin and mucosal infections [52].
3. Polymyxin B combined with a cationic porphyrin derivative demonstrated significant antibacterial activity. The cationic porphyrin derivative, a positively charged porphyrin that enhances interaction with negatively charged bacterial membranes, was conjugated with polymyxin B to create a potent antimicrobial agent. This conjugate exhibited effective bacterial eradication, even after washing, with minimal light exposure required to photoinactivate the concentrated bacterial inocula. The cationic porphyrin derivative’s ability to selectively target bacteria reduces the risk of resistance associated with antibiotic-only treatments. This approach highlights the potential of synergistic combinations of photosensitisers (PSs) and antibiotics, emphasising the versatility and promise of photodynamic therapy (PDT) as an effective and resistance-reducing strategy against *P. aeruginosa* infections [53].

### 4.4. PDT Associated with NPs

NPs play a significant role in the advancement of PDT by improving the efficacy of PS. Some approaches involving NPs include the following:

1. Incorporation of PS into Polymeric NPs: PS can be incorporated into polymeric NPs, providing a stable and targeted platform for the efficient delivery of photosensitising agents. This approach helps overcome the limitations of solubility and bioavailability of PS.
2. PS Attached to the Surface of NPs: PS can be attached to the surface of NPs, allowing for specific and targeted interactions with the target cells. This approach aims to improve the selectivity and effectiveness of PDT. PS Close to NPs: Some strategies exploit the physical proximity of PS to NPs, enhancing their therapeutic effects. These strategies may involve physical proximity without direct connection but with beneficial interactions for the effectiveness of PDT [54].
3. NPs as Photosensitisers (PS): Certain nanoparticles (NPs) can act as photosensitisers, generating photodynamic reactions when exposed to light. Their effectiveness depends on their absorbance spectra. For instance, NPs absorbing in the near-infrared (NIR) range, such as carbon nanotubes and gold, can penetrate deeper into tissues and minimise scattering, offering advantages over traditional visible light PDT. NPs with high photothermal conversion efficiency, like CNTs and polypyrrole, enhance therapeutic outcomes by converting light into heat effectively. Thus, selecting NPs with appropriate absorbance spectra is crucial for optimising both photodynamic and photothermal therapies [55].
4. Photothermal Therapy (PTT): In addition to PDT, NPs are used in PTT. In this context, near-infrared (NIR) laser irradiation is used to generate heat through the mediation of photoabsorbing agents, resulting in the denaturation of proteins, membrane rupture, and degradation of the genetic material of target cells.
5. Microemulsions (MEs): MEs have been reported to improve the efficiency of PDT by overcoming the limitations associated with the use of aqueous media to disperse photosensitising agents. They consist of two phases (aqueous and organic), with the organic phase stabilised by surfactants. Eucalyptus oil was used to destabilise the cell wall, allowing for greater PS penetration and synergistic effects [56].
6. Gold-Based NPs (AuNPs): AuNPs, including smaller gold nanoclusters, have received considerable attention owing to their photoactivatable properties, excellent biocompatibility, and ease of surface functionalisation. They can be used for both PDT and PTT, generating singlet oxygen under NIR light excitation and exhibiting photothermal properties when combined with organic dyes, such as indocyanine green [52]. These approaches highlight the diversity of strategies that utilise NPs to improve the efficacy and specificity of PDT in diverse biomedical applications.

### 4.5. PDT Delivered via Hydrogels

Hydrogels are a class of water-expanded three-dimensional polymer networks with tunable physicochemical properties that satisfy specific requirements under different conditions [9]. As promising materials, they have been extensively applied in the biomedical field, from studies on physiological and pathological mechanisms to tissue regeneration and disease therapy [9].

Hydrogels have been extensively studied as matrices for biomedical applications due to their ability to crosslink under mild conditions, excellent biocompatibility, and adjustable biochemical and biophysical properties [10]. Because the structure and properties of hydrogels closely mimic the microenvironment of many human tissues, they are widely employed in various biomedical applications [10].

**Figure 2:**
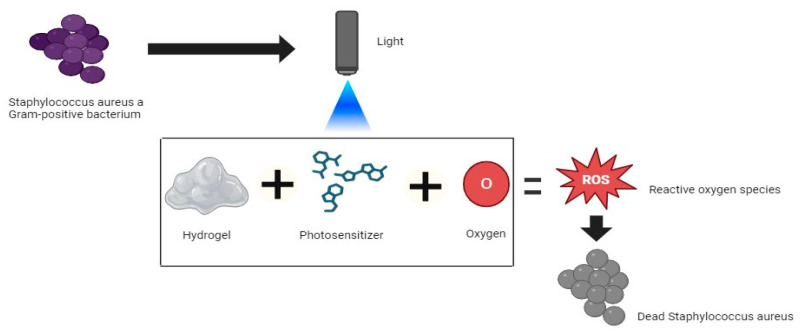
Mechanism of photodynamic therapy combined with hydrogel for combatting *Staphylococcus aureus*. Created with BioRender.com.

Hydrogels have demonstrated good performance as cell carriers in various clinical applications [57]. Loading various antibacterial agents onto hydrogels is an efficient strategy for enhancing antimicrobial effects. To prevent the emergence of drug-resistant bacteria, phototherapeutic strategies, such as the use of hydrogels loaded with radiofrequency + 405 nm LED irradiation, have been widely used for antibacterial applications [8]. PDT using hydrogels and different types of PSs (Figure 2) has been reported to exhibit antibacterial activity against *S. aureus* in several studies [5,12,13,14].

### 4.6. Application of PDT in Biofilm and Its Usefulness In Vivo

Biofilms represent a highly resistant and complex form of bacterial organisation composed of a matrix of exopolysaccharides (EPSs) that protects bacteria against various external stimuli. This structure provides considerable resistance, making treatment, including PDT, more challenging [44]. The following are some important aspects of biofilms.

1. EP Matrix: The EPS matrix forms a three-dimensional structure surrounding the bacterial cells. In some cases, the matrix is composed of polysaccharides, proteins, and metal ions. The presence of metal ions can confer a neutral or polyanionic charge on the matrix, depending on the predominant type of EP [58].
2. Resistance to External Aggression: The biofilm acts as a protective barrier, providing resistance to bacteria against external aggression, such as the host immune response, medications, and other antimicrobial agents. The matrix can trap antimicrobials, preventing them from reaching bacterial cells [58].
3. Greater Resistance Compared to Planktonic Cells: The biofilm formation confers significantly greater resistance, estimated to be between 10 and 1000 times greater than that of planktonic bacterial cells. This feature makes biofilms challenging to eradicate [59].
4. Chemical Signalling and Bacterial Cooperation: Biofilm formation involves chemical signalling between bacteria, allowing the coordination of surface adherence and cell differentiation. This bacterial cooperation results in the creation of a complex and organised microbial community [44].
5. Protection from Environmental Fluctuations: The matrix protects against environmental fluctuations, such as changes in humidity, temperature, and pH. Furthermore, the concentration of nutrients is favoured, and waste can be efficiently eliminated.
6. Challenges for PDT: In PDT, the presence of a biofilm represents a challenge, as the matrix limits the diffusion of PSs into the bacterial plasma membrane, leading to a reduction in the production of singlet oxygen. Specific strategies must be developed to overcome these barriers and make PDT effective against bacteria in biofilms [59].
7. Understanding the complexity of biofilms is crucial for developing effective therapeutic approaches, particularly in clinical situations where persistent biofilm-based infections are common.

## 5. Conclusions

This systematic review indicates that photodynamic therapy (PDT) delivered through hydrogels can effectively inhibit the growth of *S. aureus* bacteria and biofilms in vitro. Hydrogels, due to their controlled release and localised application capabilities, show potential advantages over traditional methods of delivering photosensitisers. However, it is important to note that PDT is primarily effective for treating local infections, rather than systemic infections, due to limitations in light penetration and photosensitiser activation. While hydrogels offer significant promise, they are not the only method for delivering photodynamic agents. Other delivery systems, such as nanoparticles and microemulsions, also hold potential and should be considered. There is a critical need for comparative studies to evaluate the efficacy of PDT using different delivery methods.

The review highlights a significant gap in the current research: the lack of in vivo studies assessing the effectiveness of PDT in treating *S. aureus* infections, particularly those caused by antibiotic-resistant strains. Future research should focus on conducting in vivo studies using appropriate animal models to confirm the efficacy and safety of PDT. Additionally, there is a need for research to explore how different PDT delivery methods can be adapted for clinical trials. In summary, while PDT with hydrogels shows potential, further research is essential to validate these findings in vivo, explore alternative delivery methods, and develop protocols for translating hydrogel-based PDT into clinical practice. Future studies should also investigate the effectiveness of PDT against multidrug-resistant microorganisms and address the limitations associated with photosensitisers in such contexts.

## Figures and Tables

**Table 1:** Database and search strategies.

Database	Search Strategy	Results
PubMed	((((“Anti-Infective Agents” [Mesh] OR Anti Infective Agents OR Antiinfective Agents OR Anti-Infective Agent OR Anti Infective Agent OR Microbicides OR Anti-Microbial Agent OR Anti Microbial Agent OR Antimicrobial Agents OR Anti-Microbial Agents OR Anti Microbial Agents OR Microbicide OR Antimicrobial Agent) AND (“*Staphylococcus aureus*” [Mesh])) AND (“Gram-Positive Bacterial Infections” [Mesh] OR Gram Positive Bacterial Infections OR Gram-Positive Bacterial Infection)) AND (“Photochemotherapy” [Mesh] OR Photochemotherapies OR Photodynamic Therapy OR Photodynamic Therapies)) AND (“Hydrogels” [Mesh] OR Hydrogel OR In Situ Hydrogels OR In Situ Hydrogel OR Patterned Hydrogels OR Patterned Hydrogel)	09
Scopus	(“Hydrogels” OR “Hydrogel” OR “In Situ Hydrogels” OR “In Situ Hydrogel” OR “Patterned Hydrogels” OR “Patterned Hydrogel”) AND (“Photochemotherapy” OR “Photochemotherapies” OR “Photodynamic Therapy” OR “Photodynamic Therapies”) AND (“Gram-Positive Bacterial Infections” OR “Gram Positive Bacterial Infections” OR “Gram-Positive Bacterial Infection”) AND (“*Staphylococcus aureus*”) AND (“Anti-Infective Agents” OR “Anti Infective Agents” OR “Antiinfective Agents” OR “Anti-Infective Agent” OR “Anti Infective Agent” OR “Microbicides” OR “Anti-Microbial Agent” OR “Anti Microbial Agent” OR “Antimicrobial Agents” OR “Anti-Microbial Agents” OR “Anti Microbial Agents” OR “Microbicide” OR “Antimicrobial Agent”)	25
Web of Science	((((ALL = (Anti-Infective Agents OR Anti Infective Agents OR Antiinfective Agents OR Anti-Infective Agent OR Anti Infective Agent OR Microbicides OR Anti-Microbial Agent OR Anti Microbial Agent OR Antimicrobial Agents OR Anti-Microbial Agents OR Anti Microbial Agents OR Microbicide OR Antimicrobial Agent)) AND ALL = (*Staphylococcus aureus*)) AND ALL = (Gram-Positive Bacterial Infections OR Gram Positive Bacterial Infections OR Gram-Positive Bacterial Infection)) AND ALL = (Photochemotherapy OR Photochemotherapies OR Photodynamic Therapy OR Photodynamic Therapies)) AND ALL = (Hydrogels OR Hydrogel OR In Situ Hydrogels OR In Situ Hydrogel OR Patterned Hydrogels OR Patterned Hydrogel)	03

**Table 2:** Information from each study.

Authors	Country	Methods	What Was Analysed?	Conclusion
Chen, C. P., et al., 2015 [12]	Taiwan	Toluidine blue O (TBO) hydrogel = TBO + chitosan + HPMC. Irradiation = 100 J/cm^2^ at 635 ± 20 nm.	The photodynamic efficacy of the hydrogel was tested in vitro against *Staphylococcus aureus* biofilms. Confocal laser scanning microscopy was used to assess the penetration of TBO into viable solutions. Adding HMPC improved the physicochemical properties of the chitosan hydrogel, such as hardness, viscosity, and bioadhesion; however, higher HMPC concentrations led to a decreased antimicrobial effect.	The ideal bioadhesive formulation for topical antimicrobial PDT will need to balance the desired drug release rate and the mechanical properties of the formulation, as these factors influence clinical effectiveness and ease of application. The penetration of the TBO biofilm depends on the physicochemical properties of the HTC hydrogel.
Liang, H., et al., 2017 [13]	China	TBO as photosensitiser. TBO hydrogel or light alone (630 nm) showed no antibacterial activity against *S. aureus*.	A new TBO hydrogel was developed for periodontitis treatment, utilising carbomer as the base and NaOH as the neutraliser. TBO hydrogel formulations have been employed as on-demand drug delivery systems for clinical treatments. The antibacterial activity of PDT using TBO hydrogel was tested against *S. aureus*. These TBO hydrogel formulations were optimised using response surface methodology.	A TBO hydrogel was developed for photodynamic therapy against *S. aureus*, yielding better results than PDT with an aqueous TBO solution. The hydrogel released 50% of TBO within 4 h and 68.26% within 24 h. Over six weeks, the TBO hydrogel maintained consistent colour, transparency, pH, and viscosity when stored at 4 °C, 25 °C, and 40 °C. The hydrogel or light alone showed no antimicrobial effect on *S. aureus*; only the combination of light with the TBO hydrogel exhibited antibacterial activity.
Mao, C., et al., 2017 [14]	China	Hydrogel = Ag/Ag@AgCl/ZnO nanostructures. Method = UV light reduction, ZnO added via NaOH precipitation. Irradiation = 300 W xenon lamp (visible light).	A hydrogel composite incorporating carboxymethyl cellulose and Ag/Ag@AgCl/ZnO hybrid nanostructures was developed. This composite demonstrates outstanding photocatalytic activity and broad antibacterial efficacy against Gram-positive bacteria when exposed to visible light.	Leveraging the generation of reactive oxygen species, the system demonstrated significantly improved photocatalytic activity, extensive antibacterial effects against *S. aureus* (a Gram-positive bacterium), and accelerated wound healing. The hydrogel system featured controlled and sustained release of Ag^+^ and Zn^2+^, facilitated by reversible swelling and shrinking in response to pH changes, highlighting its substantial potential for tissue repair and antibacterial applications.
Zheng, Y., et al., 2019 [5]	China	Laser = 630 nm diode (5 mW, 4 mW/cm^2^, 23 mm periotip). Photosensitiser = toluidine blue.	In vitro antibacterial tests against *S. aureus* utilised response surface methodology to optimise the TBO hydrogel formulation. The stability, pH, and antibacterial activity of the TBO hydrogel remained consistent at 4 °C, 25 °C, and 40 °C over a 6-week period. Additionally, the TBO combined with a carbomer hydrogel demonstrated release rates of 51.28% after 4 h and 69.80% after 24 h.	The optimal TBO hydrogel formulation consisted of 0.5% (*w*/*v*) carbomer 934, a TBO concentration of 0.01 mg/mL, 0.5% (*v*/*v*) ethanol, 0.5% (*v*/*v*) Tween 80, and a NaOH to carbomer mass ratio of 0.4 (*w*/*c*). The hydrogel’s properties, including appearance, clarity, viscosity, antibacterial activity, and pH, remained stable at 4 °C, 25 °C, and 40 °C for up to 6 months. It effectively inhibited Propionibacterium acnes, *S. aureus*, and *Escherichia coli*. These results indicate that the new TBO hydrogel is promising for acne treatment, with further studies needed to assess cellular toxicity and conduct animal trials.
Du, P., et al., 2023 [8]	China	Hydrogel = G-quartets + riboflavin (photocatalyst). Function = photodynamic antibacterial therapy with H_2_O_2_ production. Irradiation = 450 nm, emission recorded from 500–600 nm.	A photoactive supramolecular material based on G-quartets was developed. This material is self-assembled from guanosine (G) and 4-formylphenylboronic acid/1,8-diaminooctane, with riboflavin incorporated as a photocatalyst into the G4 nanowires for post-irradiation photodynamic antibacterial therapy. The G4 materials, which exhibit hydrogel-like properties, act as a scaffold for the riboflavin and guanosine reductant, facilitating the photo-triggered production of therapeutic H_2_O_2_.	Supramolecular G4 materials loaded with riboflavin, exhibiting gel-like properties, were demonstrated as a proof-of-concept for post-irradiation antibacterial therapy of infected wounds. These G4 hydrogels acted as dressing materials, structurally incorporating riboflavin through covalent bonding and aromatic stacking, while providing guanosine as a reductant to reduce photoexcited riboflavin and facilitate O_2_ reduction to generate H_2_O_2_. The hydrogels, after irradiation, showed strong antibacterial activity, effectively killing Gram-positive bacteria, Gram-negative bacteria, and multi-drug-resistant bacteria both in vitro and in vivo, with biosafety and no significant cytotoxicity. The riboflavin-loaded G4 hydrogels achieved a sterilisation rate greater than 99.999% against *S. aureus, E. coli*, and methicillin-resistant *S. aureus*, and demonstrated excellent antibacterial efficacy in infected rat wounds.
Elkihel, A., et al., 2023 [15]	France	Hydrogel = Xylan-TCPP with varying PS/Xylan ratios. Irradiation = white LED light for 5 h (25 J/cm^2^). Photosensitiser = meso-TCPP.	The antimicrobial activity of the hydrogels was evaluated under visible light irradiation against two strains of Gram-positive bacteria, *S. aureus* and *Bacillus cereus*. The preliminary results demonstrated notable effectiveness against these bacteria, suggesting that these hydrogels hold significant potential for treating bacterial skin infections of these species using photodynamic antimicrobial chemotherapy.	Xylan-based hydrogels containing photosensitisers were developed using TCPP as a crosslinker. Swelling tests revealed that the xyl-TCPP-3 hydrogel, which contained the smallest amount of TCPP, exhibited favourable swelling properties. Preliminary antibacterial tests against two strains of Gram-positive bacteria indicated that this hydrogel showed photodynamic activity only when exposed to light. The covalent attachment of TCPP to the Xylan component appears to reduce the photosensitiser’s toxicity in the absence of light. However, the concentration of TCPP required for effective photosensitisation seems to be higher than what is typically reported in the literature.
Zheng, Y., et al., 2023 [16]	China	Hydrogel = Au25Capt18 in carrageenan. Function = dual-mode antibacterial effects (PTT + PDT). Irradiation = NIR light at 808 nm.	Natural polysaccharide carrageenan embedded in atomically precise gold nanoparticles was reported as a novel hydrogel platform for PTT and PDT antibacterial therapy triggered using single infrared light.	Atomically precise gold nanocluster-embedded hydrogels were developed by crosslinking Au25Capt18 with carrageenan, serving as an effective photothermal and photodynamic agent for antibacterial applications with single near-infrared (NIR) laser irradiation. The contribution of photothermal therapy (PTT) to antibacterial efficacy was found to be more substantial than that of photodynamic therapy (PDT) in the Au25Capt18 hydrogels. In vivo studies demonstrated that these hydrogels could effectively eliminate pathogenic bacteria and promote the healing of infected wounds.

## Data Availability

The data presented in this study will be provided without restrictions upon communication with the corresponding author.
